# Revisiting the Curtis Procedure for Boutonniere Deformity Correction

**Published:** 2015-07

**Authors:** Lee Seng Khoo, Vasco Senna-Fernandes

**Affiliations:** Ivo Pitanguy Institute, Rua Dona Mariana 65, Botafogo, Rio De Janeiro, Brazil

**Keywords:** Curtis Procedure, Boutonniere Deformity, Correction

**DEAR EDITOR**

Boutonnière deformity (BD) is a debilitating deformity where the proximal interphalangeal (PIP) joint of the finger is flexed, and the distal interphalangeal (DIP) joint is hyperextended.^1^^-^^3^ BD is commonly seen after injury and also as a sequela of progressive inflammatory conditions such as rheumatoid arthritis.^1^^-^^3^ Various techniques have been described to address the imbalance between the extensor and flexor mechanism to correct BD including (i) Salvi repositioned the lateral bands dorsally,^4^ (ii) Tenotomy of the extensor tendon distal to the triangular ligament was described for chronic BD,^4^ (iii) Littler and Eaton dissected out the extrinsic and interosseous tendons from the lumbrical and oblique retinacular ligaments and centralized the lateral bands,^5^ (iv) Stack utilized the superficial flexor tendon to reconstruct the central slip and redistributed the forces across the PIP,^6^ (v) Matev used the lateral band on one side to reconstruct the central slip, elongating it on the contralateral side to make use of a single lateral band,^7^ and (vi) Hou et al. described a method of reconstructing the central slip by employing autologous palmaris longus tendon.^8^

*Anatomy*

Injury to the extensor tendon prevents the finger from active extension at the PIP joint. The three components to the extensor tendon injury includes central slip rupture, triangular ligament attenuation and lateral band volar migration.^1^^-^^3^ As the deformity progresses, the now dominant flexor superficialis creates constant flexion at the PIP joint. Initially, the DIP joint exhibits an extensor lag. Over time, the lumbrical and interosseous muscles (intrinsics) lose their insertion into the middle phalanx due to an incompetent central slip. Their force of action is diverted entirely through the lateral bands. The lateral bands then get displaced volarly and contract. This is accompanied by secondary shortening of the oblique retinacular ligaments (ORL). Together, these changes cause hyperextension at the DIP joint in addition to the already hyperflexed PIP joint.^1^^-3^

The authors revisit the Curtis staged procedure to correct BD.^9 ^The Curtis procedure first was described by Curtis et al in 1983 allowing a staged redistribution of forces to attain a more anatomically functional finger.^9^ The procedure has a unique advantage that the surgical steps are carried out stepwise and ends when desirable correction is achieved on table.^9^

*Indications/Contraindications*

All patients should be subjected to a minimum of 1 month splinting prior to attempting surgical treatment. A variety of splints such as the Bunnel safety pin splint or dynamic spring based splits are available. As success of surgical treatment is dependent upon the degree of joint contracture prior to reconstruction; it is imperative that adequate passive extension can be elicited before embarking on surgical treatment.^9^

Laboratory work up should be done for infective and inflammatory markers. Standard view radiographs of the hand and digit, including posteroanterior, oblique, and lateral are mandatory. 

The Haines-Zancolli test is helpful to aid in a decision of either conservative or surgical treatment. This test tests the structures around the PIP joint. The PIP joint is held in a neutral position while the DIP joint is flexed by the examiner. If the DIP joint does not flex, the retinacular (collateral) ligaments or proximal interphalangeal capsule are tight. If the PIP joint is flexed and the DIP joint flexes easily, the ORL are tight and the capsule is normal. During the test, the patient remains passive and does no active movements.^9^ The test result is considered negative if passive flexion of the DIP joint is still possible with the PIP joint maintained in extension .The test result is positive if flexion of the DIP joint is not possible with the PIP joint in extension. A positive Haines-Zancolli test result equates poor outcome with conservative treatment.^9^

*Technique*

There are four stages described in the Curtis procedure to correct BD.^9^ Full passive mobility of the PIP joint must be elicited and also documented preoperatively prior to performing the Curtis procedure. The surgery is carried out under local anesthesia to allow the surgeon to test for active extension at each stage. It is not necessary to perform all four stages of the Curtis procedure and the operation stops when full extension is obtained at any stage. Patient is prepped and draped with the operated arm abducted at 90 degrees^9^ (Figure1).

**Fig. 1:**
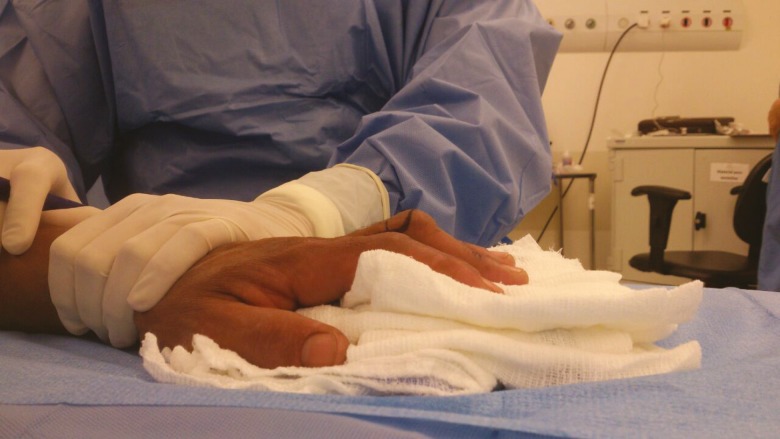
Lateral view of Boutonniere’s deformity of left middle finger

Stage I entails a lazy “S” incision made over the PIP joint (Figure 2 and 3). The transverse retinacular ligament is located and mobilized in both the distal and proximal aspects (Figure 4 and 5). The transverse retinacular ligament originates from the flexor tendon sheath in the PIP joint and inserts on the lateral edges of the conjoined lateral bands.^9^

**Fig. 2:**
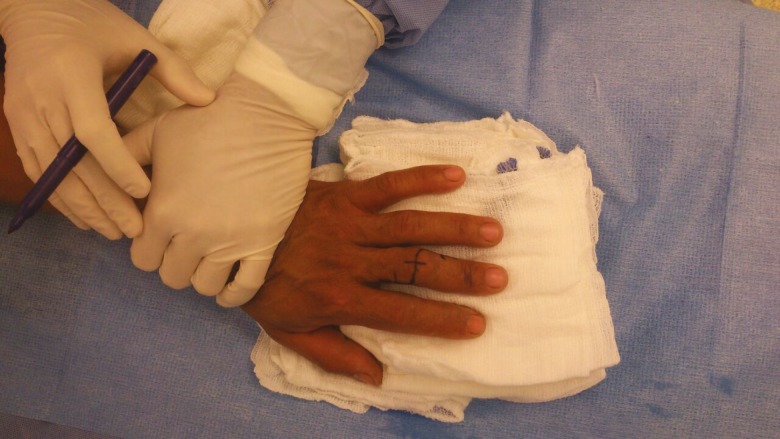
Lazy S marking over the PIP joint

**Fig. 3:**
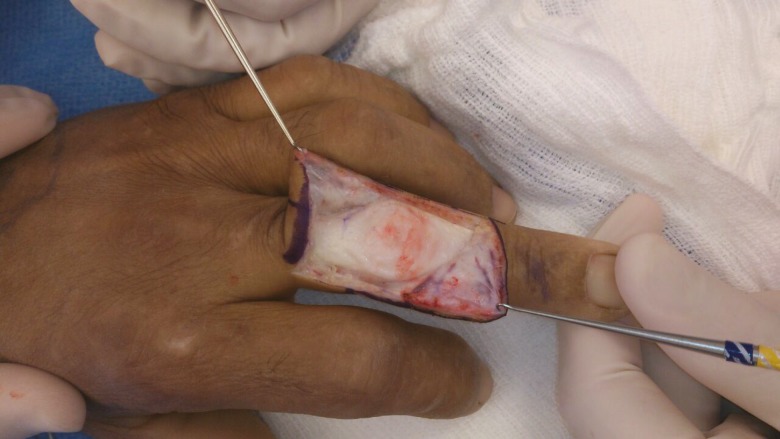
The flaps are raised and reflected

**Fig. 4:**
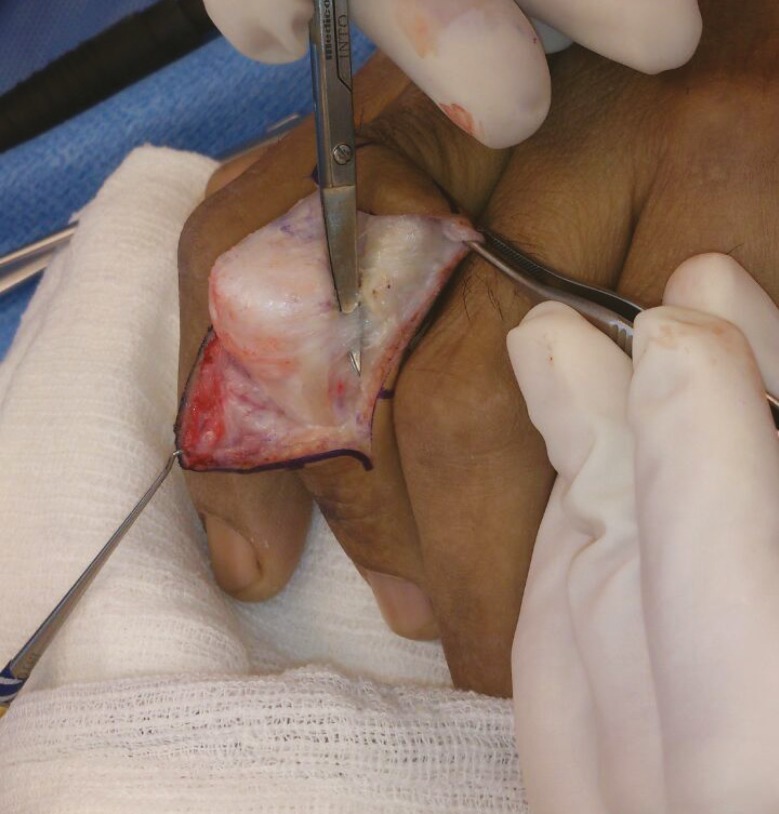
The transverse retinacular ligament on the right

**Fig. 5:**
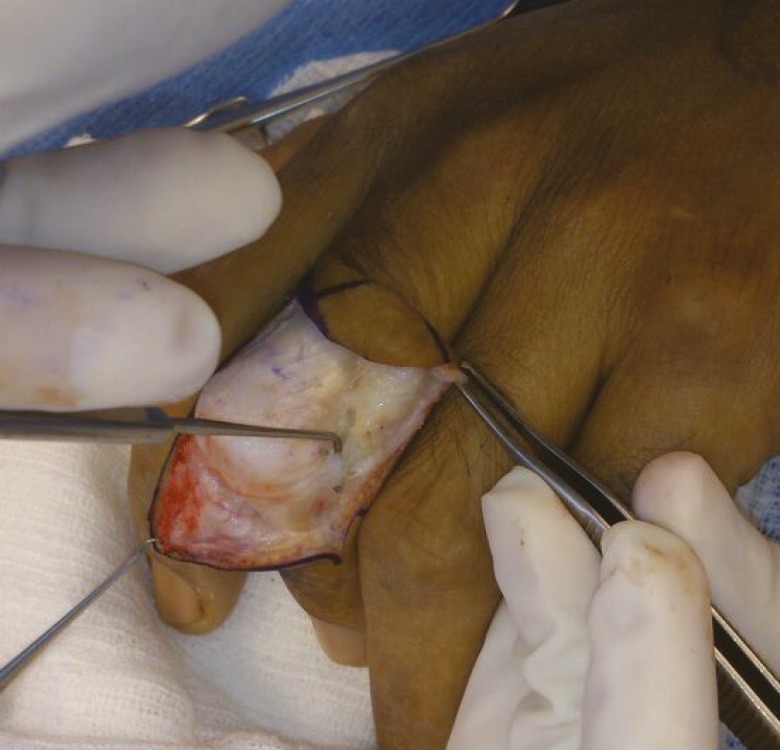
The transverse retinacular ligament on the left

Two lateral bands on each digit arise proximal to the PIP from the Extensor Digitorum Communis (EDC). The lateral bands travel on either side of proximal phalanx, and continue around the lateral aspect of PIP joints and finally both slips join together in a triangular aponeurosis to form the extensor tendon to the digital phalanx. The lateral bands are also formed from the deep head of the dorsal interossi combining with the volar interossi. They insert onto the base of the distal phalanx to extend the DIP joint.^9^

In short the lateral bands are held in place over the PIP by the transverse retinacular ligament. Tenolysis of the extensor tendon is then carried out after freeing the transverse retinacular ligament. If full extension is obtained at Stage I, the operation stops here. If full extension is not obtained in Stage I, the transverse retinacular ligament (TRL) is then transected, and this maneuver allows the lateral bands to swing dorsally. If full extension is elicited, the metacarpal joint can then be splinted at 70 degrees of flexion, the PIP and DIP joints are splinted at 0 degrees for 1 week, and then dynamic PIP joint splinting is started. Dynamic PIP mobilization splint is made of spring steel piano wire with coils. The DIP is immobilized with a palmar splint to prevent DIP hyperextension.^9^

If a 20-degree or smaller lag persists after stage II, a Fowler tenotomy is performed. The extensor mechanism distal to the triangular ligament is transected obliquely. The triangular ligament spans the two lateral bands, preventing them from subluxing volarly. It is important to note that terminal tendon tenotomy is performed and not central slip tenotomy (Figure 6). The rationale is to create a “Mallet finger” thereby decreasing the tone of the DIP joint and allowing the extensor mechanism to slide proximally to increase the extensor tension at the PIP joint. If full extension is present by this stage, the operation stops.^9^

**Fig. 6:**
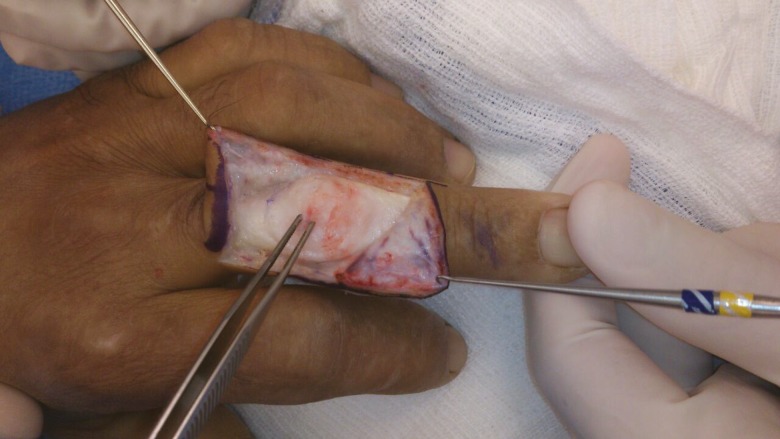
The central slip is identified. In this case Fowler’s tenotomy was not performed

However if an extensor lag of more than 20 degrees is still evident after stage II, Curtis advocated proceeding directly to stage IV. The central slip is dissected free (Figure 7) and advanced about 4 to 6 mm into a drill hole in the dorsal base of the middle phalanx (Figure 8). The lateral bands, which are no longer tight are loosely sutured to the central tendon.^9^

**Fig. 7:**
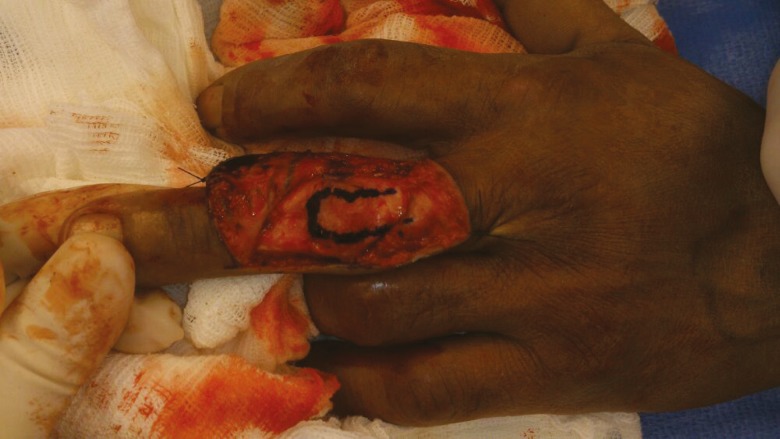
Marking of the central slip with methylene blue dye

**Fig. 8:**
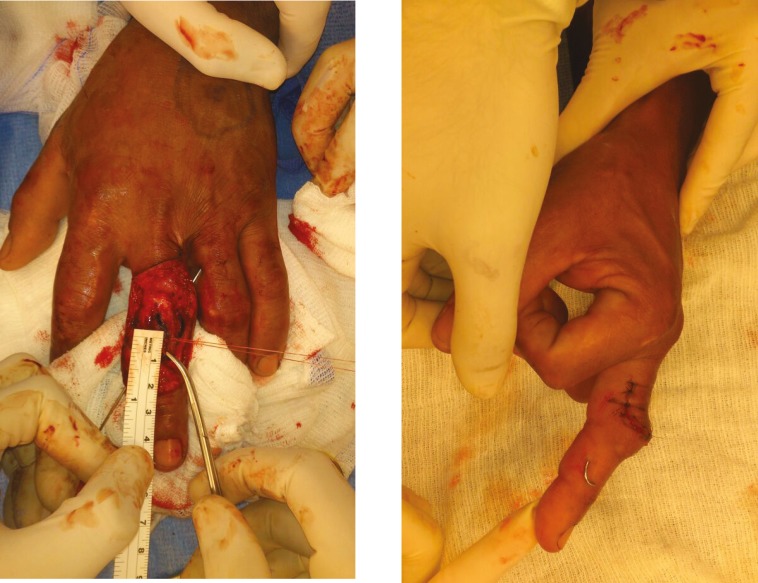
The central slip is raised and advanced 4-6mm into drill hole of dorsal base of middle phalanx

The skin can then be closed with 4-0 or 5-0 non absorbable sutures and dressing applied. All patients need protection of the PIP post surgery. Fixation with a K-wire immobilizes the PIP (Figure 9 and 10), followed by some form of splinting. The length of time of K-wire fixation and splinting depends on the initial injury, the stage of the procedure performed, and the surgeon’s preference. Active DIP joint motion is mandatory in the postoperative period.^9^

**Fig. 9:**
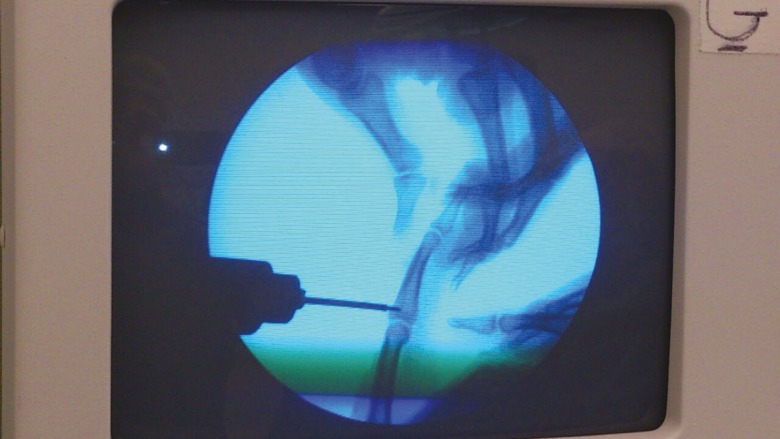
Immobilization with K-wire confirmed via C-arm

**Fig. 10:**
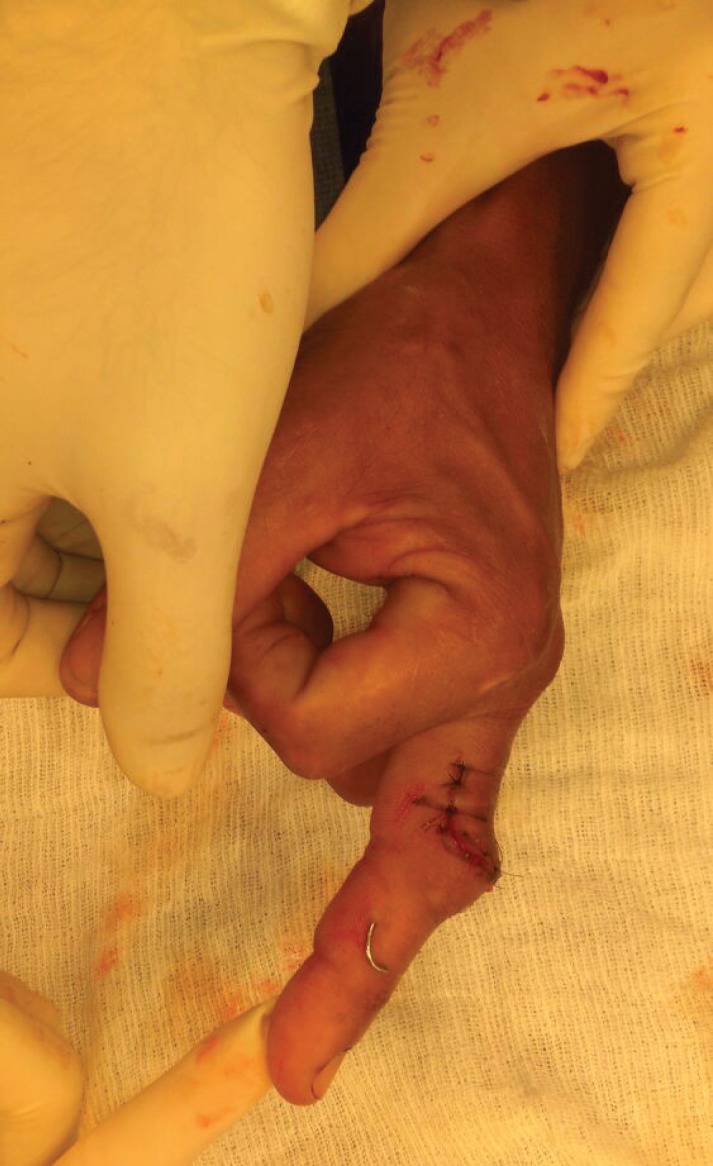
Final appearance of finger after completion of Curtis procedure

*Expected Outcomes*

A 10-degree IP joint lag with an average 31-degree improvement from the preoperative state was reported by Curtis at 1 year follow up.^9^ Patients who underwent stage IV reconstruction revealed an average PIP joint preoperative lag of 55 degrees, a 17-degree lag persisted postoperatively.^9^ Our own experience based on 18 cases operated using the Curtis procedure with 7 requiring Stage IV reconstruction; 9 obtained full restoration of DIP joint extension. All 7 patients requiring Stage IV reconstruction achieved near normal DIP joint extension with a residual 10 degree lag on average. In rheumatoid boutonniere deformity hand function only deteriorates when the deformity is severe. Therefore the surgical procedures used should not risk or sacrifice existing function. The staged Curtis procedure allows the surgeon to stop at the stage where adequate result is obtained.^9^

*Complications*

Possible complications of the Curtis procedure are (i) Failure of reconstruction with relapse requiring reoperative treatment, (ii) Infections although rare; may need open drainage and antibiotics, (iii) Loss of digital motion, resulting in a totally stiff finger and (iv) Complex regional pain syndrome, often debilitating and chronic.^9^

To expedite recovery and to prevent a relapse in the early postoperative period, patients need to be educated of the importance of maintaining the PIP in a proper splint for the prescribed amount of time following repair. The more modern dynamic splints used toward the end of mobilization may shorten the splinting duration needed in the postoperative period.

## CONFLICT OF INTEREST

The authors declare no conflict of interest.

## Note

Please cite this paper as:

Khoo LS, Senna-Fernandes V. Revisiting the Curtis Procedure for Boutonniere Deformity Correction. World J Plast Surg 2015;4(2):180-184.
